# Topical administration of a doxorubicin-specific monoclonal antibody prevents drug-induced mouth apoptosis in mice

**DOI:** 10.1054/bjoc.2001.2188

**Published:** 2001-12

**Authors:** A Balsari, C Rumio, D Morelli, L Sfondrini, E Nardini, I Barajon, S Ménard

**Affiliations:** Institute of Pathology, Department of Human Anatomy, Faculty of Medicine, Milan University, Milan, Italy; Dept of Experimental Oncology, Istituto Nazionale per lo Studio e la Cura dei Tumori, Via Venezian 1, Milan, 20133, Italy

**Keywords:** mucositis, doxorubicin, monoclonal antibody, apoptosis, antidotal activity

## Abstract

One of the most severe side effects of anti-tumour chemotherapy is mucositis due to drug toxicity for rapidly dividing cells. We show here that anti-DXR monoclonal antibodies can prevent DXR-induced damage. Indeed, apoptosis, confined to the proliferative compartment of the basal mucosa, observed in the tongue of DXR-treated mice was completely inhibited by topical application of the anti-DXR antibodies. © 2001 Cancer Research Campaign http://www.bjcancer.com


*British Journal of Cancer* (2001) **85**(12), 1964–1967
© 2001 Cancer Research Campaign
doi: 10.1054/ bjoc.2001.2188, available online at http://www.idealibrary.com on IDEAL

### Topical administration of a doxorubicin-specific monoclonal antibody prevents drug-induced mouth apoptosis in mice

**A Balsari[1], C Rumio[2], D Morelli[3], L Sfondrini[3], E Nardini[3], I Barajon[1] and S Ménard[3]**

[1]Institute of Pathology and [2]Department of Human Anatomy, Faculty of Medicine, Milan University, Milan, Italy; [3]Dept of Experimental Oncology, Istituto Nazionale per lo Studio e la Cura dei Tumori; Via Venezian 1, 20133 Milan, Italy

**Summary** One of the most severe side effects of anti-tumour chemotherapy is mucositis due to drug toxicity for rapidly dividing cells. We show here that anti-DXR monoclonal antibodies can prevent DXR-induced damage. Indeed, apoptosis, confined to the proliferative compartment of the basal mucosa, observed in the tongue of DXR-treated mice was completely inhibited by topical application of the anti-DXR antibodies. © 2001 Cancer Research Campaign http://www.bjcancer.com

**Keywords**: mucositis, doxorubicin, monoclonal antibody, apoptosis, antidotal activity

Antineoplastic agents are currently not highly specific for cancer cells and also damage normal cells. Indeed, chemotherapy affects virtually every organ system in the body to an extent that depends on the number of dividing cells (Goodman, 1989). Thus, tissues that typically exhibit rapid cell turnover are the most sensitive to the toxic effect of chemotherapy. Squamous epithelial cells of gastrointestinal mucosa divide rapidly, with a cell generation time of 12 to 24 h, and are therefore particularly vulnerable to the cytotoxicity of antineoplastic agents. The disruption in cell division and maturation becomes histologically evident within 5 days of drug exposure. As the damaged cells slough, the mucosa becomes thin and denuded, accompanied by delayed cellular renewal, mucosal atrophy, inflammation and often ulceration. The incidence and severity of stomatitis or oral mucositis are highly dependent on the therapy, i.e, the drug used, total dosage, drug schedule, and concomitant therapy, and on the patient, i.e., tumour type and age (Sonis, 1983). Patients who receive drugs such as DXR or methotrexate can experience intense stomatitis. Patients with hematological malignancies develop stomatitis two to three times more frequently than do patients with solid tumors (Dreizen, 1978). Younger patients in whom cell renewal occurs more rapidly are at greater risk for stomatitis, although they also heal more quickly for the same reason.

Grade 1 or 2 stomatitis usually resolves by 3 weeks, in time for the next course of treatment. Moderate or severe (grade 3 or 4) stomatitis that has not fully resolved at the time of retreatment usually suggests not only a delay in treatment until the mucosa has healed, but also a reduction in the drug dose. Moreover, stomatitis in patients with severe granulocytopenia can lead to widespread hematogenous bacterial dissemination since the oral lesions serve as entry for the oral microflora to the bloodstream (Sonis, 1985). Hospitalization is often necessary to maintain body hydration, to control pain, and to prevent or manage infection.

*Received 9 May 2001*
*Revised 27 July 2001*
*Accepted 20 September 2001*

*Correspondence to:* S Ménard

Of the approximately 50 drugs used in chemotherapy regimens, DXR (Adriamycin®) has found one of the widest applications, because of its activity against a broad range of malignancies. DXR acts primarily at the DNA level through intercalation. The ability of this drug to form covalent adducts with DNA has been correlated to cellular apoptosis in tumor models (Zeman et al, 1998). The pathogenesis of gastrointestinal injury induced by DXR has been associated with the increase in intestinal apoptotic cells (Sun et al, 1998). We have derived a murine monoclonal antibody (Mab) against DXR which also reacts with other anthracycline derivatives (Balsari et al, 1990). Topical application of this Mab to the skin of rats prevents DXR-induced alopecia (Balsari et al, 1994). Moreover oral administration of the anti-DXR Mab prevents chemotherapy-induced gastrointestinal toxicity in mice (Morelli et al, 1996).

In the present study, we show that anti-DXR monoclonal antibodies, topically administered 2 h before i.v. injection of a high dose of DXR, completely block DXR-induced oral apoptosis in an animal model. Thus, anti-drug antibodies might provide a prophylactic oral treatment to maintain the integrity of the oral mucosa.

### METHODS

### Mice

Six- to eight-week-old female C57BI/6 mice were obtained from Charles River (Calco, Italy). All mice were treated in accordance with Institutional guidelines. Before sacrifice, mice were anaesthetized with 0.2 ml/20 g body weight of 10 mg/ml ketamine and 0.05% xylazine.

### Reagents

Doxorubicin hydrochloride (Adriamycin) was supplied by Pharmacia & Upjohn SpA (Milan, Italy). A fresh solution of DXR was prepared just before use. The anti-DXR Mab MAD11 (IgG2a) selectively recognizes epitopes located at or near the aromatic D ring of the anthracycline molecule (Balsari et al, 1990). Ascitic fluids contained about 1 mg/ml of specific or unrelated antibody. Human immunoglobulins (Sandoglobulina, Sandoz, Milan, Italy) were used in immunohistochemistry experiments to avoid the background reactivity on murine tissue due to the use of biotinylated anti-murine IgG antibodies.

### Immunohistochemistry

To evaluate immunoglobulin oral absorption, 200 μl of human IgG solution 50 mg/ml (Sandoz) were gently applied on oral mucosa of 3 mice using a micropipette. Two hours later mice were sacrificed, tongues and cheeks removed and immersed in polyethylene-glycol, frozen in vapor nitrogen and stored at –80˚C. The samples were separated into 10 μm parasagittal sections with a freeze microtome at –25˚C and transferred to microscope slides.

Sections were rehydrated with 1% bovine serum albumin in PBS for 15 min at room temperature, fixed in 4% paraformaldehyde for 5 min at 4˚C and incubated overnight at 4˚C with anti-human IgG peroxidated donkey IgG (Dako A/S, Glostrup, Denmark) diluted 1:20.

After rinsing in PBS, sections were blocked for endogenous peroxidase activity with 0.3% hydrogen peroxide–PBS solution for 40 min and incubated with diaminobenzidine (Sigma Chemical Co., St. Louis, MO) for 5 min. The samples were observed with a Nikon Eclipse 300 equipped with interference contrast.

### Induction of apoptosis by DXR

Three groups of mice (3 animals/group) were treated i.v. with 16–22–30 mg/kg body weight of DXR respectively. Four hours after treatment the animals were sacrificed, tongue removed and immersed in polyethylene-glycol, frozen in vapor nitrogen and preserved at –80˚C. The samples were parasagittal sectioned with a cryostat obtaining 10 μm thick sections and examined by TUNEL assay, as described by Gavrieli et al (1992). Briefly, terminal deoxyribonucleotyl-transferase (Boehringer Mannheim, Mannheim, Germany) was used to insert FITC-conjugated nucleotides (Boehringer Mannheim) at the 3′ ends of the DNA. POD converter was used to detect FITC conjugated nucleotides and the peroxidase was developed with diaminobenzidine (Sigma) for 5 min. The samples were observed with a Nikon Eclipse 300 equipped with interference contrast.

### Effects of Mab MAD11 on DXR-induced apoptosis

Ascitic fluid (400 μl), containing anti-DXR Mab MAD11 or unrelated antibody, were gently applied to the oral mucosa of mice (3 animals/group) using a micropipette. Two or 24 h later the animals were treated i.v. with 30 mg/kg body weight of DXR. At 4, 8 and 24 h after DXR treatment mice were sacrificed, tongue removed and examined by TUNEL as described above.

### RESULTS

To verify oral mucosa permeability to immunoglobulin penetration, human immunoglobulins were gently painted onto murine oral mucosa, and the painted tissue removed, sectioned and immunohistochemically examined 2 h later. High level absorption of immunoglobulins was observed on murine tongue, mainly at the level of the tunica submucosa (Figure 1). By contrast, essentially no immunoglobulin absorption was observed in the cheek mucosa

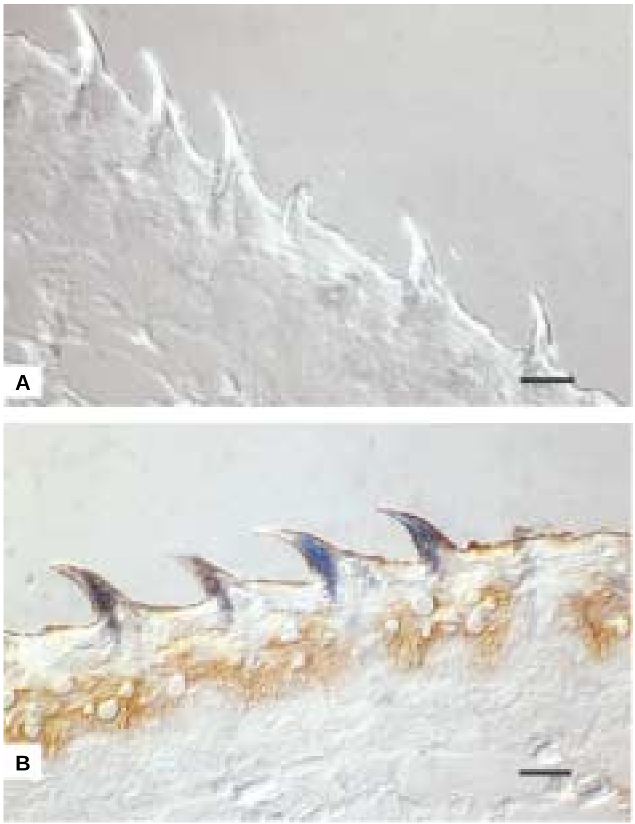

**Figure 1** Interference contrast photomicrographs of a parasagittal section of murine tongue gently painted with saline buffer (**A**) or human immunoglobulins (**B**). Bar 50 μm

(data not shown), probably due to the high keratinization of this tissue in mice.

DXR induces cytotoxic effects on mouse small intestine cells, predominantly in the base of the crypt. Typical apoptotic changes are observed, such as nuclear and cytoplasmic condensation and preservation of organelles in the early stages (Thakkar et al, 1992; Anilkumar et al, 1992). To evaluate DXR-induced apoptosis in the oral cavity, mice were treated i.v. with DXR (16, 22 or 30 mg/kg body weight), sacrificed 4 h later, and tongues were analyzed by TUNEL and microscopy. No apoptotic cells were observed by TUNEL on tongues obtained from mice treated with DXR at 16 or 22 mg/kg, whereas at 30 mg/kg numerous basal layer cells of the tongue exhibited a positive nuclear staining indicative of apoptosis (Figure 2). Apoptotic cells were observed in specialized gustative and common epithelia. No morphological alterations in sections of tongue were observed at that time by microscopy, even at the dose of 30 mg/kg body weight of DXR (data not shown).

When 400 μl of anti-DXR Mab MAD11 was gently applied to murine tongue 2 h before i.v. injection of DXR (30 mg/kg body weight), drug-induced apoptosis was completely prevented, as evaluated 4, 8 and 24 h after anthracycline treatment (Figure 3B–3C). On the contrary apoptotic cells were present on tongues obtained from mice treated with the unrelated antibody (Figure 3A). When anti-DXR Mab MAD11 was applied 24 h before DXR treatment, partial or no protection was observed (data not shown), indicating either inactivation of the antibodies or dilution in surrounding tissues.

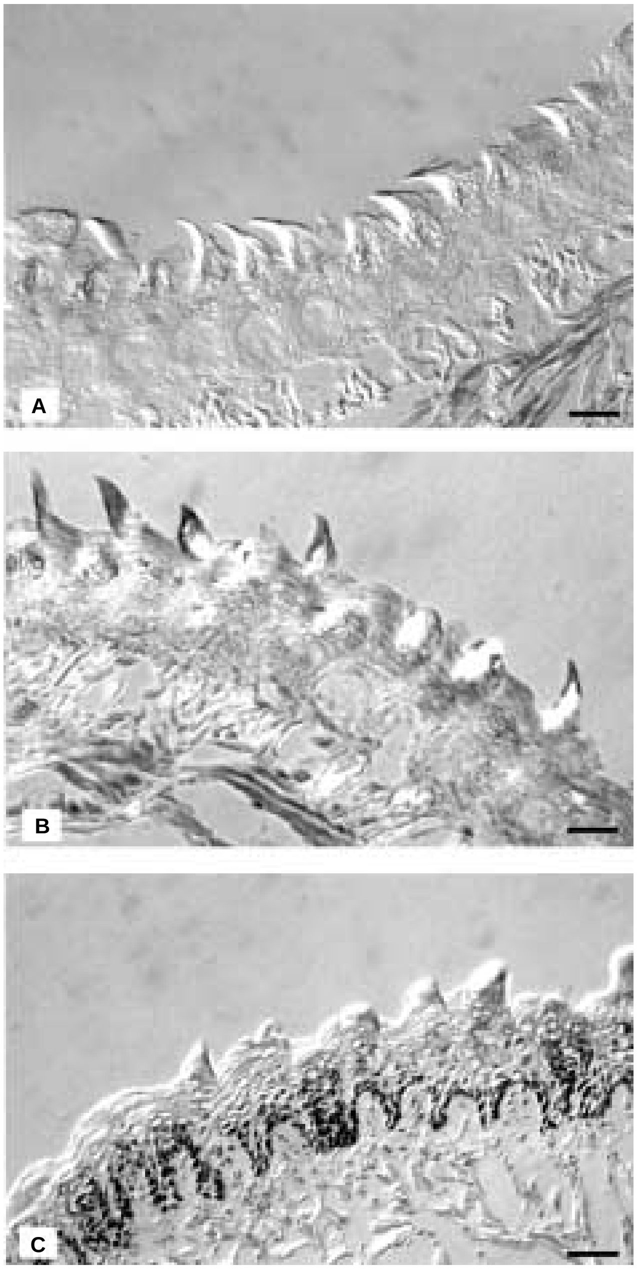

**Figure 2** Interference contrast photomicrographs of parasagittal sections of murine tongue illustrating apoptosis induced by DXR. Mice were treated i.v. with 16 mg/kg (**A**), 22 mg/kg (**B**) and 30 mg/kg (**C**). Bar 85 μm

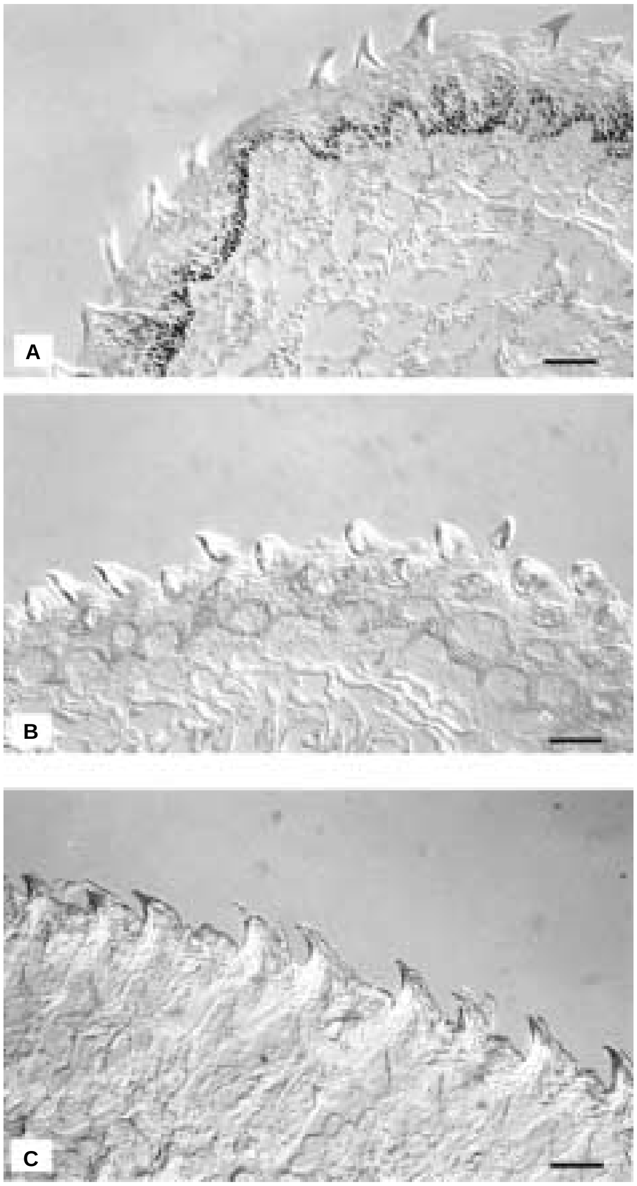

**Figure 3** Photomicrographs of parasagittal sections of murine tongue illustrating protection from DXR-induced apoptosis. Mice were orally treated with unrelated antibody (**A**), or with anti-anthracycline antibody (**B**–**C**), and 2 h later i.v. injected with 30 mg/kg of DXR. The tongues were evaluated 4 (**A**–**B**) or 24 (**C**) h later. Bar 100 μm

### DISCUSSION

Histological examination of tongue samples from mice treated with 30 mg/kg of DXR revealed numerous apoptotic cells, mainly confined to the proliferative compartment of epithelium, which is not surprising since cell proliferation occurs predominantly in the basal two-thirds of the mucosa.

DXR doses that induce cell apoptosis in the tongue are higher than those that induce apoptosis in the intestinal mucosa (Morelli et al, 1996). The higher responsiveness of intestinal mucosa is perhaps due to the intestinal recycling of the drug that increases the quantity of drug delivered to individual intestinal cells. Because of the impossibility to evaluate mucositis in mice (Sonis et al, 1990), apoptosis was considered as the end point for the toxic activity of DXR. Indeed only hamster cheek pouch seems to be sensitive to DXR-induced mucositis; in this model, modulation of drug-induced mucositis was observed by topical administration of transforming growth factor β that reduces cheek epithelial cell proliferation (Sonis et al, 1994).

Painting of the tongue with anti-DXR antibodies completely inhibited DXR-induced apoptosis. The tongue surface is particularly absorptive in general (Oyama et al, 1999; Purushotham et al, 1995), so that a high concentration of blocking antibodies can be delivered by topical administration. The efficacy of antibody administration at 2 h but not at 24 h before drug treatment indicates the rapid dilution or degradation of the reagent in the mucosa. On the other hand, the protection seems to be permanent since it was observed also at 24 h from DXR injection.

*© 2001 Cancer Research Campaign*

Chemotherapy with high dose of anthracycline commonly induces painful and dose-limiting oral mucositis. Oral treatment with anti-DXR antibodies appears to be appropriate for patients who develop mucositis after the first cycle of DXR therapy for almost any tumor. The exception is oral cancer, where antibody treatment might inhibit not only mucositis but also the drug activity on the tumor cells. No side effects are expected from oral administration of immunoglobulins, as suggested by many other observations (Tollemar et al, 1999; Johansson et al, 1999).

In conclusion, simple oral administration of anthracycline antibody to prevent anthracycline-induced oral apoptosis may allow a larger number of patients to tolerate high-dose chemotherapy.

### ACKNOWLEDGEMENTS

This work was partially supported by CNR/Biotecnologie and AIRC.

### REFERENCES

Anilkumar TV, Sarraf CE, Hunt T and Alison MR (1992) The nature of cytotoxic drug – induced cell death in murine intestinal crypts. *Br J Cancer* **65**: 552–558

Balsari A, Morelli D, Ménard S, Tagliabue E, Colnaghi MI and Ghione M (1990) A new monoclonal antibody recognizing anthracyclinic molecule. *Anticancer Res* **10**: 129–132

Balsari AL, Morelli D, Ménard S, Veronesi U and Colnaghi MI (1994) Protection against doxorubicin-induced alopecia in rats by liposome-entrapped monoclonal antibodies. *FASEB J* **8**: 226–230

Dreizen S (1978) Stomatotoxic manifestations of cancer chemotherapy. *J Prosthetic Dentistry* **40**: 650–655

Gavrieli Y, Sherman Y and Ben-Sasson SA (1992) Identification of programmed cell death in situ via specific labeling of nuclear DNA fragmentation. *J Cell Biol* **119**: 493–501

Goodman M (1989) Managing the side effects of chemotherapy. *Semin Oncol Nurs* **5**: 29–52

Johansson JE and Ekman T (1999) Gut mucosa barrier preservation by orally administered IgA-IgG to patients undergoing bone marrow transplantation: a randomised pilot study. *Bone Marrow Transplant* **24**: 35–39

Morelli D, Ménard S, Colnaghi MI and Balsari A (1996) Oral administration of anti-doxorubicin monoclonal antibody prevents chemotherapy-induced gastrointestinal toxicity in mice. *Cancer Res* **56**: 2082–2085

Oyama Y, Yamano H, Ohkuma A, Ogawara K, Higaki K and Kimura T (1999) Carrier-mediated transport systems for glucose in mucosal cells of the human oral cavity. *J Pharm Sci* **88**: 830–834

Purushotham KR, Offenmuller K, Bui AT, Zelles T, Blazsek J, Schultz GS and Humphreys-Beher MG (1995) Absorption of epidermal growth factor occurs through the gastrointestinal tract and oral cavity in adult rats. *Am J Physiol* **269**: G867–G873

Sonis ST (1983) Epidemiology, frequency, distribution, mechanisms, and histopathology. In *Oral complications of cancer chemotherapy*, Peterson D and Sonis S (eds), pp 1–20 New York: Nijhoff

Sonis ST (1985) Adverse effects of treatment: oral complications of cancer therapy. In *Cancer principles and practice of oncology*, Devita VT, Hellman S and Rosenberg S (eds), pp 2014–2021, Philadelphia: Lippincott

Sonis ST, Lindquist L, Van Vugt A, Stewart AA, Stam K, Qu G-Y, Iwata KK and Haley JD (1994) Prevention of chemotherapy-induced i = ulcerative mucositis by transforming growth factor b3. *Cancer Res* **54**: 1135–1138

Sonis ST, Tracey C, Shklar G, Jenson J and Florine D (1990) An animal model for mucositis induced by cancer chemotherapy. *Oral Surg Oral Med Oral Pathol* **69**: 437–443

Sun Z, Wang X, Wallen R, Deng X, Du X, Hallberg E and Andersson R (1998) The influence of apoptosis on intestinal barrier integrity in rats. *Scand J Gastroenterol* **33**: 415–422

Thakkar NS and Potten CS (1992) Abrogation of adriamycin toxicity in vivo by cycloheximide. *Biochem Pharmacol* **43**: 1683–1691

Tollemar J, Gross N, Dolgiras N, Jarstrand C, Ringden O and Hammarstrom (1999) Fungal prophylaxis by reduction of fungal colonization by oral administration of bovine anti-Candida antibodies in bone marrow transplant recipients. *Bone Marrow Transplant* **23**: 283–290

Zeman SM, Phillips DR and Crothers DM (1998) Characterization of covalent adriamycin-DNA adducts. *Proc Natl Acad Sci USA* **95**: 11561–11565

*© 2001 Cancer Research Campaign*
